# Delayed Bitterness of Citrus Wine Is Removed Through the Selection of Fining Agents and Fining Optimization

**DOI:** 10.3389/fchem.2019.00185

**Published:** 2019-04-02

**Authors:** Jingying Bi, Hua Li, Hua Wang

**Affiliations:** ^1^College of Enology, Northwest A & F University, Yangling, China; ^2^Ningxia Polytechnic, Ningxia Vocational Education Park, Yinchuan, China

**Keywords:** citrus wine, delayed bitterness, limonoid, fining agent, agar, gelatin

## Abstract

“Delayed bitterness” (DB) in citrus wine is caused by limonoids and determines the acceptability to consumers. In this study, a variety of fining agents, specifically gelatin, agar, chitosan, bentonite, the crosslinking agent polyvinylpyrrolidone (PVPP), diatomite, and casein, were evaluated for their ability to lower DB in citrus wine. Factorial experiments with three factors at four levels (L${}_{3}^{4}$) and with two factors at three levels (L${}_{2}^{3}$) were used to determine the optimal effect. We found that a mixture of agar (125 mg/L) and gelatin (30 mg/L) not only decreased the limonoid concentration and clarified the liquor, but also increased the precipitation content, retention rate of ascorbic acid, and antioxidant capacity. After treatment, the quality of the citrus wine was improved, and a few volatile chemical compounds were lost. We determined that agar and gelatin were the best fining agents for reducing DB in citrus wine.

## Introduction

Citrus (*Citrus reticulata Blanco*) represent the highest-value fruit crop in the world and the third largest contributor to the trade of agricultural products (Wu et al., 2006). According to the Food and Agriculture Organization of the United Nations (FAO), 17,848.22 million tons of citrus fruit was produced in the world in 2015, accounting for 27% of the world's total fruit production (FAO, 2016). Citrus is popular for its nutritious quality, distinctive flavor, and various bioactive compounds with antioxidant, antiallergic, antimicrobial, antiviral, anticancer, and antineoplastic properties (Roy and Saraf, 2006; Vieira et al., 2009). However, the processing of citrus products involves formidable problems, such as “delayed bitterness” (DB), which affects acceptability. DB is caused by a group of oxygenated tetranortriterpenoid compounds, named limonoids, which are commonly found in species from the Rutaceae, Meliaceae, and Cneoraceae families, and less frequently, from the Simaroubaceae family (Champagne et al., 1992; Roy and Saraf, 2006). As a salt of limonoic acid, A-ring lactone is found in fruit tissues; however, when the fruit is crushed, juiced, and frozen, the combined action of the juice acids and the enzyme limonin-D-ring hydrolase (EC 3.1.1.36) converts the tasteless A-ring lactone to limonin, which is bitter (Maier et al., 1977, 1980; Puri, 1990; Berhow et al., 2000). Previous data revealed that the taste threshold level of limonin is 1 ppm in distilled water and 6 ppm in juices (Guadagni et al., 1973). Further, nomilin is thought to have only a minor contribution to the bitter taste of orange juice (Rouseff, 1982). Therefore, using appropriate technology in citrus processing is important to reduce bitterness in citrus juices below a threshold level for consumer acceptability. Although several chemical, physical, and microbiological methods have been devised and proposed to remove limonoids from citrus juice, very few of these methods have practical application. This is because de-bittering is achieved at a cost of nutritional quality, for example, through a reduction in the levels of ascorbic acid, naringin, hesperidin, or total phenolic compounds (Lee and Kim, 2003; Kranz et al., 2011) and negative effects on color, flavor, taste, texture, and stability.

Fining agents are routinely used at different stages of the winemaking process to counteract constituents that are considered to adversely affect juice or wine quality. The fining process relies on adding substances that induce flocculation and settling in turbid wine, or in wine with colloidal instability. A broad range of fining agents is available for use, and fining is a common practice in winemaking (Jackson, 2014).

The purpose of this study was to investigate different concentrations of several fining agents—namely gelatin, agar, chitosan, bentonite, PVPP, diatomite, and casein—as single factors, three factors at four levels (L${}_{3}^{4}$), and two factors at three levels (L${}_{2}^{3}$) in factorial-optimization experiments to evaluate their capacity to remove limonoids from citrus wine, which has not been previously reported. Our aim was to fundamentally test the hypothesis that specific fining agents are suitable to reduce the levels of DB compounds in citrus wine. To that end, three experiments were conducted: (a) a preliminary screening of seven fining agents at different concentrations to identify those that reduce DB levels in citrus wine; (b) testing of a subset of fining agents via L${}_{3}^{4}$ orthogonal trial on limonoid removal and turbidity reduction in citrus wine; and (c) final testing and optimization of the best-performing fining agents using a L${}_{2}^{3}$ experiment.

## Experimental

### Fruit Materials

Six-years old navel orange trees (*Citrus sinensis* L. Osbeck), representing the major cultivated variety among the different ecological types of citrus in China, approximately 2.5–3.0 m tall, from the Huacharm Co., Ltd. orchard located in Qu County, Sichuan, China (~30°85′N, 106°95′E) were chosen for this study. All fruits were harvested at commercial maturity, based on size, color, shape, weight, and the absence of physical injuries or infections. Navel oranges with a °Brix maturity index of 11.90%, total sugar (as glucose) of 58.46 g/L, titratable acidity (TA; as citric acid) of 7.20 g/L, a pH of 3.63, and a juice rate of 41.72% were hand-picked in the middle of December 2016 and immediately transported to the College of Enology at Northwest A&F University.

### Chemicals

Standard limonin and 2,6-dichloroindophenol were purchased from Yuan Ye Bio-Tech Co., Ltd (Shanghai, China); 1,1-diphenyl-2-picrylhydrazyl (DPPH), 2,2′-azino-bis(3-ethylbenzothiazoline-6-sulphonic acid) (ABTS), the 73 aroma standards and HPLC-grade methanol and acetonitrile were purchased from Sigma–Aldrich (Beijing, China). Ultra-pure water was obtained using a Milli-Q water purification system (Millipore, Bedford, MA, USA). For the winemaking processes, agar, gelatin, chitosan, bentonite, PVPP, casein, and diatomite was purchased from McLean Bio-Tech Co., Ltd. (Shanghai, China), and yeast extract powder was obtained from Angel Yeast Co., Ltd. (Yichang, China).

### Winemaking Process

Citrus wine samples were obtained in relatively small quantities (20 L) (Li, 2002). The peels of navel oranges were removed manually ensuring that the fruits were intact. The fruits were squeezed to separate the juice from the pulp and seeds. Then, citrus juice was sulfited by the addition of 100 mg/L potassium metabisulfite (corresponding to approximately 50 mg/L sulfur dioxide) in a glass bottle (20 L capacity). The juice obtained was treated with 20 mg/L of pectolytic enzyme preparation and stored for 48 h below 10°C. The supernatant was adjusted to a temperature of 14–18°C before inoculation with 200 mg/L of active dry yeast powder. Sugar was added to increase the alcohol content to 12–13% (v/v) during the vigorous fermentation period, and the fermentation temperature was maintained between 18 and 20°C. The entire fermentation process lasted for 9–11 days. When residual sugar in the fermentation liquid dropped below 2 g/L, the wine was racked, sulfur dioxide (approximately 50 mg/L) was added, and the bottle was sealed for 10–14 days and subjected to aging. The citrus wine was subjected to different clarification treatments during its biological aging for one-and-a-half years.

The wine was analyzed for its °Brix (8.20%) and found to have a total sugar (as glucose) of 1.91 g/L, a TA (as citric acid) of 9.30 g/L, an alcohol content of 12.87% (v/v), and a pH of 3.47.

### Fining Agents and Clarification Experiments

Unclarified citrus wine was treated with different concentrations of the following seven fining agents, namely gelatin (15, 30, 45, 60, and 75 mg/L), agar (25, 50, 75, 100, 125, and 150 mg/L), chitosan (200, 400, 600, 800, and 1,000 mg/L), bentonite (200, 400, 600, 800, and 1,000 mg/L), PVPP (20, 40, 60, 80, and 100 mg/L), diatomite (250, 500, 750, 1,000, 1,250, and 1,500 mg/L), and casein (150, 300, 450, 600, 750, and 900 mg/L), all of which were mixed well. Testing of each fining agent was performed at dosages commonly used in current winemaking practices (Li et al., 2012). Aliquots (100 mL each) of wine samples were poured in colorimeter tubes and a single fining agent was added to each tube, followed by incubation in the dark at 4°C for 120 h. All fining agent treatments were conducted in triplicate and included a control check wine (CK) that contained no fining agent. The supernatant was collected and analyzed for the limonoid concentration, turbidity, titratable acidity, pH, DPPH, ABTS, and optical density at 450, 520, 570, and 630 nm (Pérez-Caballero et al., 2003). The remaining sample was centrifuged at 3,000 × g for 10 min and dried for the determination of constant weight and precipitation.

### Physicochemical Index Determination

Turbidity, pH, and total sugar, titratable acidity, pH, alcohol, ascorbic acid, and precipitation contents were analyzed according to methods commonly used in the citrus industry (Kimball, 1991). Thermal stability was assessed after fining by filtering the citrus wine into a 250 mL volumetric flask containing iodine, using a wet and qualitative medium-speed filter. The sample was incubated in a thermostatic water bath for 72 h at 67–68°C, and observations were made twice a day. If no precipitation was observed within 72 h, the samples were considered to be heat stable (Wang et al., 2015). These measurements were all performed in triplicate.

### Extraction and Determination of Limonoid Equivalents

Limonoids were selectively isolated from citrus juice via chloroform (CHCl_3_) extraction (Andrew et al., 2007). The citrus wine samples were centrifuged at 1,500 × g for 10 min at 10°C. A 1:2 mixture of clarified wine (1 mL) and CHCl_3_ (2 mL) was transferred to a test tube, followed by ultrasonication (30 min), vortexing (2 min), and centrifugation at 1,500 × g for 10 min at 10°C to expedite the phase separation. Chloroform extracts were combined, and the solvent was removed at 40°C under slow and controlled nitrogen flow. Samples were reconstituted in 0.5 mL acetonitrile. Extractions were conducted in triplicate.

### Measurement of Limonoids by Spectrophotometry

Limonoids were measured by spectrophotometry using the method of Abbasi et al. (2005). Limonin absorbance measurements were performed using a Cary 60 UV-visible spectrophotometer (Agilent Technologies, Inc., USA). Two milliliters of the acetonitrile phase was transferred to a test tube containing 5 mL of Ehrlich's reagent consisting of reagent A and reagent B (0.05 mL each). Reagent A contained 125 mg of 4-(dimethylamino)benzaldehyde, 65 mL of sulfuric acid, and 35 mL of absolute ethyl alcohol, which had been mixed and cooled, whereas reagent B consisted of 9.0 g of ferric trichloride dissolved in distilled water to a volume of 100 mL. The mixture was kept at an ambient temperature for 20 min (Appendix in Supplementary Material) to gain maximum red color. Absorbance of the upper phase was assayed at 491 nm (Appendix in Supplementary Material).

### ABTS and DPPH Analysis

The ABTS assay was based on the method of Re et al. (1999). ABTS was dissolved in water to reach a concentration of 7 mM. ABTS radical cations (ABTS^∙1^) were produced by mixing 5 mL of the ABTS stock solution with 88 μL of potassium persulfate (140 mM) in the dark at 15°C for 12–16 h. The blue-green ABTS^∙1^ solution was filtered through nylon syringe filters prior to use. To test the citrus wine, the ABTS^∙1^ solution was diluted with ethanol to an absorbance at 734 nm of 0.70 (± 0.02) and equilibrated to room temperature. An aliquot (0.1 mL) of the sample solution was added to 3.9 mL ABTS^∙1^ (A_734nm_ = 0.70 ± 0.02), followed by an 8 min reaction in the dark. Absorbance was measured using a Cary 60 UV-visible spectrophotometer (Agilent Technologies) at 734 nm.

The spectrophotometric method employed for DPPH analysis is based on the free radical DPPH-scavenging activity determined using the method described by Gadow et al. (1997). Briefly, 1 mL of freshly prepared DPPH solution (0.2 mM DPPH in ethanol) and 1 mL of sample solution were mixed, followed by a 30 min reaction in the dark. Absorbance was measured with a Cary 60 UV-visible spectrophotometer (Agilent Technologies) at 517 nm.

Appropriate solvent blanks were run in each assay. All determinations were carried out at least three times, and in triplicate, on each occasion and for each separate concentration for the standard and samples.

### Chromaticity and Color Analysis

Citrus wine samples were centrifuged and filtered through 0.45-μm filters prior to analysis. The color values of the wine were evaluated using CIELab color space (Pérez-Caballero et al., 2003) and were measured with a Cary 60 UV-visible spectrophotometer (Agilent Technologies) at four wavelengths (450, 520, 570, and 630 nm). The values L^*^ (lightness), a^*^ (from red to green), and b^*^ (from blue to yellow) (Pérez-Caballero et al., 2003), as well as C^*^ (chroma), H^*^ (hue-angle), and E^*^ (color difference) (Ramos-Escudero et al., 2012), were calculated according to the following six formulae

(1) $$\begin{array}{rcl} {\text{L}}^{*} & = & 116\times \left[{\left(\text{Y}/{\text{Y}}_{10}\right)}^{1/3}-0.1379\right] \end{array}$$

(2) $$\begin{array}{rcl} {\text{a}}^{*} & = & 500\times \left[{\left(\text{X}/{\text{X}}_{10}\right)}^{1/3}-{\left(\text{Y}/{\text{Y}}_{10}\right)}^{1/3}\right] \end{array}$$

(3) $$\begin{array}{rcl} {\text{b}}^{*} & = & 200\times \left[{\left(\text{Y}/{\text{Y}}_{10}\right)}^{1/3}-{\left(\text{Z}/{\text{Z}}_{10}\right)}^{1/3}\right] \end{array}$$

(4) $$\begin{array}{rcl} \text{C}* & = & \sqrt{{\left(a*\right)}^{2}+{\left(b*\right)}^{2}} \end{array}$$

(5) $$\begin{array}{rcl} {\text{H}}^{*} & = & \left[\text{Arctan}\left({\text{b}}^{*}/{\text{a}}^{*}\right)\right] \end{array}$$

(6) $$\begin{array}{rcl} {\text{E}}^{*} & = & {\left[{\left({\text{L}}^{*}\right)}^{2}+{\left({\text{a}}^{*}\right)}^{2}+{\left({\text{b}}^{*}\right)}^{2}\right]}^{1/2} \end{array}$$

### Volatile Chemical Compound (VCC) Analysis

Solid-phase micro-extraction gas chromatography–mass spectrometry (SPME–GC–MS) was used for VCC analysis. The experimental conditions employed were in accordance with those described by Wang et al. (2017), with minor differences.

#### SPME Analysis

All citrus wine samples were analyzed in 50 mL glass vials containing 20 mL of each sample, 2 g of NaCl, and 60 μL of the internal standard 2-octanol (0.559 mg/L). Then, magnetic agitation temperature control was needed for the vials. A magnetic stirring bar was placed in each vial, which provided agitation to the sample. Divinylbenzene/carboxen/polydimethylsiloxane (50/30 μm, StableFlex/SS [2 cm], Supelco, Bellefonte, PA., USA) was used as the solid-phase fiber for micro-extraction. SPME was performed at 40°C for 60 min before desorption of the analytes into the gas chromatograph injector.

#### GC–MS Analysis

A TRACE DSQ single quadrupole GC–MS (Thermo Finnigan, USA) system was used. For GC analysis, the analytical column consisted of a DB-Wax capillary column (30 m length, 0.25 mm inner diameter, 0.25 μm film thickness; Agilent J&W, Santa Clara, CA). He (flow rate of 1 mL/min) was used as a carrier gas. The temperature program was as follow: 40°C for 3 min, increased to 160°C at a rate of 4°C/min, raised to 230°C at a rate of 7°C/min, and held at 230°C for 8 min. The transfer line temperature was 230°C and the inlet temperature was 250°C. Mass spectra were recorded in electron impact (EI) ionization mode. The ion source temperature was 230°C. The mass range used for MS was 33–450 atomic mass units, and scanning was performed at 1 s intervals.

Identification was achieved by comparing the mass spectra obtained from the samples with those from pure standards injected under the same conditions, and by comparing the Kovats index and the mass spectra present in the Wiley MS Library Database, or in literature. The internal standard used for quantitation was 2-octanol (0.559 mg/L). Quantitative data were obtained for the identified compounds by interpolation of the relative areas vs. the internal standard area, in calibration graphs built for pure reference compounds. The concentrations of volatile compounds, for which there were no pure references, were obtained using the same calibration graphs as used for the compound with the most similar chemical structure, according to the formula and chemical character (Perestrelo et al., 2006; Li et al., 2008; Xi et al., 2011; Wang et al., 2017).

### Sensory Analysis

Sensory analysis of citrus wine was performed in duplicate according to Peng et al. (2013). Bitterness and aroma characteristics were analyzed by 15 trained panelists, aged between 21 and 38 years old (nine females and six males). The panelists were undergraduate, master's, and doctoral students of the College of Enology at Northwest A&F University, who provided informed consent to participate in the sensory tests for the present investigation, had no history of known taste disorders, and were trained in sensory experiments at regular intervals. In this study, the panelists were trained to detect citrus bitterness (mainly limonoid, nomilin, and naringin) using a 54-aroma kit (Le Nez du Vin®, France) until their accuracy of identification for each trait exceeded 95%. They were asked to describe citrus wine aroma using 4–6 terms from the aroma kit, which had been classified into different groups. In addition, the three-alternative forced-choice (3-AFC) test was used for threshold determination (Rousseff and Matthews, 1984); the panelists evaluated the citrus wine bitterness, aroma, overall impression, and other notable features of citrus wine, and graded the intensity of each characteristic using a five-point scale (1, very weak; 2, weak; 3, medium; 4, intense; 5, very intense; Glabasnia et al., 2018).

Participants signed an informed consent form and received a non-monetary gift for their participation. The participants, all non-smokers, were asked to refrain from eating, drinking, or chewing gum for 1 h prior to testing. To determine changes in the sensory properties of citrus wine samples that occurred during the fining process of eliminating limonoids, a sorting test was performed according to the sensory evaluation form of Cemeroglu (1992).

The data processed were a mixture of intensity and frequency of detection, which was calculated with the formula:

$$\begin{array}{l} MF\%=\sqrt{F\left(\%\right)I\left(\%\right)} \end{array}$$

where *F(%)* is the detection frequency of an aromatic attribute expressed as a percentage, and *I(%)* is the average intensity expressed as a percentage of the maximum intensity.

### Verification Test

To demonstrate that using 125 mg/L agar and 30 mg/L gelatin as fining agents can reduce the concentration of limonoids in citrus wine, we adopted the citrus wine model system in this study (9.0 g/L citric acid, 120 mL/L anhydrous ethanol, and 20 g/L glucose were diluted to 1 L in pure water). The pH was adjusted to 3.5 using 6 M food-grade sodium hydroxide. These model systems were designed based on the compositions of sugars and acids, and the pH in navel orange wine (from average values obtained in 2013–2017). Then, limonin (5, 10, 15, 20, and 25 mg/L) and nomilin (2, 4, 6, 8, and 10 mg/L) were added, and the optimal combination of the fining agent was verified.

### Data Analysis

All experiments were performed in triplicate. Means and standard deviations (SDs) were calculated using Excel 2007 (Microsoft, Redmond, WA, USA). Data were analyzed using Sigma Stat (SPSS Inc., Chicago, IL, USA) with significance at *P* < 0.05. Results were subjected to analysis of variance (ANOVA) and Student's *t*-test.

## Results AND Discussion

### Limonoids and Turbidity Analysis

Based on our measurements of complexes formed with limonin and Ehrlich's reagent, we determined that the wavelength of maximum absorption, time for best color, and standard curve equation were 491 nm, 20 min, and Y = 8.18779X (*R*^2^ = 0.99663), respectively. We also determined Y = 0.00110X (*R*^2^ = 0.99991) as the standard curve equation of turbidity and defined low turbidity as < 10 nephelometric turbidity units (NTUs), moderate turbidity as 10–100 NTUs, and high turbidity as over 100 NTUs.

### Single-Factor Experiment Analysis

**Figure 1:**
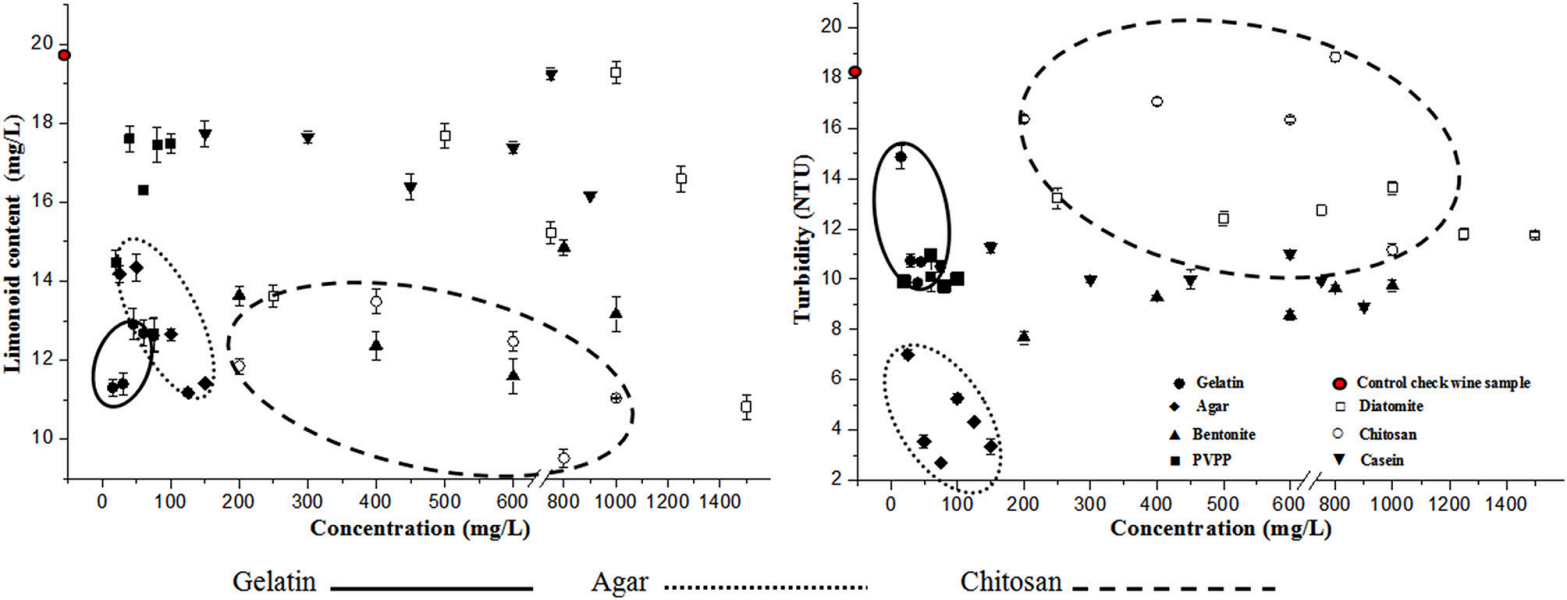
Limonoid content and turbidity of citrus wine after fining with the indicated fining agents.

Our findings from single-factor experiments clearly indicate that the limonoid content in most of the 38 samples tested was reduced after fining. Almost all of the fining agents tested removed limonoids to a certain extent from citrus wine (Figure 1). In particular, we found that a range of concentrations of gelatin, chitosan, and agar (75, 100, 125, and 150 mg/L), as well as bentonite (400 and 600 mg/L), and diatomite (1,500 mg/L) removed higher amounts of limonoids from citrus wine. In contrast, we found that the limonoid contents in samples after fining with casein (750 mg/L) and diatomite (1,000 mg/L) were comparable to that found in the CK1 (Control check wine sample for the single-factor test) sample. Similarly, we found that the turbidity of citrus wine samples decreased in most of the 38 samples tested after fining (Figure 1). All fining agents decreased the turbidity of citrus wine; particularly, certain concentrations of agar, bentonite, and PVPP (20, 40, and 80 mg/L), and casein (300, 450, 750, and 900 mg/L) markedly decreased wine turbidity. In contrast, we found the highest turbidity with chitosan (800 mg/L) and CK1, with NTU values of 18.83 and 18.29, respectively.

As shown in Figure 1, agar, gelatin, and chitosan lowered the limonoid content and turbidity of citrus wine. A combination of fining agents can more effectively control nutrition loss and improve the flavor and stability of citrus wine. In fact, the clarification effect of a combination of fining agents is better than that found using single fining agents (Wang et al., 2016). Therefore, we next designed an orthogonal L${}_{3}^{4}$ experiment to determine the optimal combination of fining agent mixtures, which might complement each other and synergistically reduce the limonoid content and turbidity further.

### L${}_{3}^{4}$ Orthogonal Experiment Analysis

Our findings from the L${}_{3}^{4}$ orthogonal experiment showed that the limonoid content in most citrus wine samples tested were reduced after fining (Table 1). Of note, we found significantly reduced limonoids with sample combination #2 (10.74 mg/L) compared to that found in CK2 (Control check wine sample for the L${}_{3}^{4}$ test) (16.59 mg/L). The least effective fining agent combination was sample combination #9, with a limonoid content of 12.49 mg/L. We also found that all tested combinations of fining agents lowered turbidity, with the greatest reduction found in sample #6 (4.89 NTUs), and the least reduction was found in sample #2 (6.59 NTUs), as compared to a turbidity of 11.53 NTUs in CK2 (Table 1). Using ANOVA, we found that the three fining agents (gelatin, agar, and chitosan) affected the limonoid content in that order. However, chitosan, a natural cationic flocculent, had significant disadvantages for further use. In addition to higher cost, adding chitosan in excess is not conducive to clarification of the wine, as it confers a specific scent to the liquor body and lightens the color of the liquor due to pigment adsorption (Luo et al., 2007). Thus, agar and gelatin were chosen as fining agents for optimization in an L${}_{2}^{3}$ experiment.

**Table 1:** Content of limonoids and turbidity of citrus wine after conducting L${}_{3}^{4}$ and L${}_{2}^{3}$ fining experiments.

**Sample no**.	**Fining agents (mg/L)**			**Results**	
	**Gelatin**	**Agar**	**Chitosan**	**Limonoids (mg/L)**	**Turbidity (NTUs)**
1	10	100	200	11.30 ± 0.46^ab^	6.37 ± 0.95^d^
2	10	125	400	10.74 ± 0.81^a^	6.59 ± 0.73^d^
3	10	150	600	11.73 ± 0.39^bc^	5.18 ± 0.32^ab^
4	20	100	400	11.66 ± 0.39^bc^	6.14 ± 0.69^cd^
5	20	125	600	11.72 ± 0.42^bc^	5.24 ± 0.31^ab^
6	20	150	200	11.83 ± 0.51^bc^	4.89 ± 0.15^a^
7	30	100	600	11.29 ± 0.71^ab^	4.91 ± 0.23^a^
8	30	125	200	12.09 ± 0.53^cd^	4.94 ± 0.44^a^
9	30	150	400	12.49 ± 0.53^d^	5.66 ± 0.33^bc^
CK2				16.59 ± 1.04^e^	11.53 ± 0.66^e^
K11	33.77	34.25	35.22		
K12	35.21	34.55	34.89		
K13	35.87	36.05	34.74		
R1	2.10	1.80	0.48	Gelatin > agar> chitosan	
K21	18.14	17.42	16.20		
K22	16.27	16.77	18.39		
K23	15.51	15.73	15.33		
R2	2.63	1.69	3.06		Chitosan > gelatin > agar
10	10	100		12.70 ± 1.14^bc^	3.75 ± 0.24^a^
11	20	100		12.44 ± 0.66^abc^	4.05 ± 0.73^abcd^
12	30	100		12.98 ± 0.87^c^	4.79 ± 0.98^cd^
13	10	125		11.95 ± 1.12^a^	4.22 ± 0.70^abcd^
14	20	125		12.27 ± 1.22^ab^	4.89 ± 0.92^d^
15	30	125		11.80 ± 1.74^a^	3.89 ± 0.15^ab^
16	10	150		12.16 ± 1.51^ab^	3.95 ± 0.44^abc^
17	20	150		13.13 ± 1.19^c^	4.42 ± 0.75^abcd^
18	30	150		12.02 ± 0.66^ab^	4.68 ± 0.46^bcd^
CK3				16.34 ± 0.58^d^	10.98 ± 0.42^e^

*1–18: Treatment with combinations of different fining agents at different concentrations. Data in the same column not sharing the same letters reflect significant differences (P < 0.05). CK3: control check wine sample for the L${}_{2}^{3}$ test. K ij: the average of all experimental results, j factors, and i levels. R1: The extreme level of limonoids in citrus wine samples treated with different concentrations of different fining agents; R2: the extreme level of turbidity in citrus wine samples treated with different concentrations of different fining agents. All data are expressed as the mean ± SD of three replicates of 20 citrus wine samples*.

### L${}_{2}^{3}$ Optimization Experiment Analysis

From our L${}_{2}^{3}$ optimization experiment, we found that limonoids of most citrus wine samples were reduced after fining (Table 1). Of these, sample combinations #15 (11.80 mg/L) and #17 (13.13 mg/L) exhibited the greatest and least reduction respectively, although all tested samples had significantly lower limonoid contents, compared to that found in CK3 (16.34 mg/L). Similarly, we found that all tested wine samples had lower turbidity after fining, with the greatest reduction found in samples #10 (3.75 NTUs) and #15 (3.89 NTUs), and the least reduction found in sample #14 (4.89 NTUs). All measures of turbidity in tested samples treated with fining agents were significantly lower than that found in CK3 (10.98 NTUs). Based on these findings, we determined that sample combination #15, consisting of 125 mg/L agar and 30 mg/L gelatin, was the best combination.

### Thermal Stability and Optimization of Fining Agents

**Figure 2:**
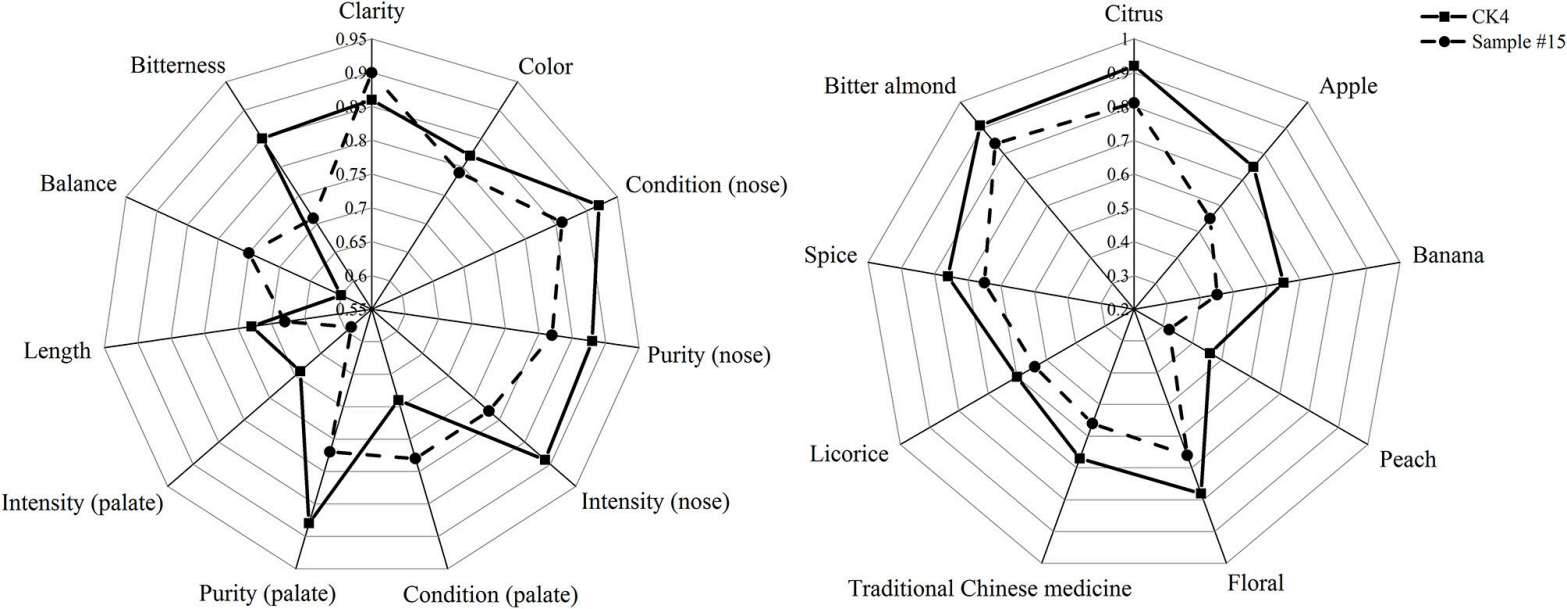
Quantitative Descriptive Analysis (QDA) of **(A)** sensory quality and **(B)** aroma of sample #15 and CK4.

We assessed the thermal stability after single-factor, L${}_{3}^{4}$, and L${}_{2}^{3}$ optimization experiments. We found that the citrus wine was light amber in color having no visible precipitation, no peculiar smell, and moderate turbidity (10–100 NTUs). We also evaluated the thermal stability based on results from our fining agent-optimization process and the identification of agar (125 mg/L) and gelatin (30 mg/L) as the optimal fining agents of citrus wine. Heat stability assessment of sample #15 was performed in comparison to CK4 (the control check wine sample) (Table 2 and Figure 2).

**Table 2:** Physicochemical composition and CIELab parameters of citrus wine after fining and the heat stability experiment (HSE).

	**CK4**	**Sample #15**	**CK4 after HSE**	**Sample #15 after HSE**
Limonoids (mg/L)	16.53 ± 0.84	10.54 ± 0.61	–	–
pH	3.43 ± 0.01	3.56 ± 0.01	–	–
TA (as citric acid) (g/L)	8.74 ± 0.10	8.15 ± 0.06	–	–
Precipitation content (g)	0	0.15 ± 0.00	–	–
Turbidity (NTUs)	11.23 ± 0.46	4.59 ± 0.17	91.68 ± 1.90	83.43 ± 4.14
Retention rate of ascorbic acid (%)	96.87 ± 1.02	32.95 ± 0.87	7.86 ± 0.12	2.93 ± 0.09
DPPH (μmol/L)	0.53 ± 0.01	0.51 ± 0.04	0.10 ± 0.01	0.06 ± 0.02
ABTS (μmol/L)	3.63 ± 0.04	3.49 ± 0.08	1.95 ± 0.08	1.66 ± 0.18
L^*^	95.61 ± 0.23	96.13 ± 0.21	78.55 ± 0.89	76.85 ± 0.45
a^*^	−1.67 ± 0.06	−1.63 ± 0.05	6.72 ± 0.48	7.46 ± 0.25
b^*^	19.74 ± 0.55	18.06 ± 0.63	50.43 ± 0.72	50.31 ± 0.35
C^*^	19.81 ± 0.54	18.13 ± 0.62	50.88 ± 0.78	50.86 ± 0.36
H^*^	−1.49 ± 0.01	−1.48 ± 0.01	1.44 ± 0.01	1.42 ± 0.00
E^*^	97.64 ± 0.12	97.83 ± 0.09	93.59 ± 0.34	92.15 ± 0.39

–: Testing was not performed. All data are expressed as the mean ± standard deviation of three replicates of four citrus wine samples. NTUs: Nephelometric turbidity units; The standard curve equations for DPPH and ABTS were Y = −0.54710X + 0.65410 (R^2^ = 0.99840) and Y = −0.69616X + 0.71117 (R^2^ = 0.99921), respectively. L^*^: lightness; a^*^: from red to green; b^*^: from blue to yellow; C^*^: chroma; H^*^: hue angle; and E^*^: color difference.

### VCC Identification

As shown in Table 3, several different VCCs were identified by SPME–GC–MS, including alcohols, esters, acids, aldehydes, and ketones. We detected 50 compounds in CK4 and 36 compounds in sample #15, with total concentrations of 2.72 × 10^3^ and 1.92 × 10^3^ mg/L, respectively. Based on our analysis, the VCCs of CK4 were clearly richer than those of sample #15 and that this difference was due to the adsorption of VCCs by the fining agent.

**Table 3:** Concentration of VCCs in CK4 and sample #15.

**No**.	**Compound**	**Retention time (min)**	**Concentration (mg/L)**		**Odor threshold^1^, ^2^ (mg/L)**	**Odor description^1^, ^2^**
			**CK4**	**Sample #15**		
**ALCOHOLS**						
1	1-Pentanol	9.95	469 ± 16	468 ± 22	64^3^	Balsamic, bitter almond^3^
2	Methionol^*^	21.82	22.2 ± 1.8	–	1^4^	Raw potato, garlic, cooked vegetable^4^
3	Phenylethanol	24.91	243 ± 10	232 ± 11	14^5^	Flowery, pollen, perfume, rose^5^
	**Subtotal**		**734**	**700**		
	**Subtotal (%)**		**27.0**	**36.5**		
**ESTERS**						
1	Ethyl acetate	3.52	266 ± 18	–	7.5^5^	Sweet, fruity^5^
2	Ethyl butanoate	5.70	69.1 ± 7.9	63.4 ± 4.0	0.02^5^	Sour fruit, strawberry, Fruity^5^
3	Isopentyl acetate	7.52	15.5 ± 1.0	14.4 ± 0.9	0.03^5^	Fresh, banana^5^
4	Ethyl hexanoate	10.44	13.6 ± 0.7	6.40 ± 0.32	0.014^5^	Green apple, fruity, strawberry, anise^5^
5	Methyl octanoate	14.42	2.74 ± 0.11	2.20 ± 0.09	0.10–0.40	Orange, intense citrus
6	Ethyl octanoate	15.78	90.9 ± 5.4	83.6 ± 3.7	0.005^5^	Pineapple, pear, floral^5^
7	Ethyl decanoate	20.47	27.4 ± 1.3	27.7 ± 1.7	0.2^5^	Fruity, fatty, pleasant^5^
8	Ethyl benzoate	20.83	46.8 ± 3.5	45.4 ± 4.4	0.053	Ripe fruit
9	Diethyl succinate	21.16	177 ± 10	178 ± 10	1.20	Fruity, grape
10	Ethyl trans-4-decenoate^*^	21.40	6.10 ± 0.29	6.11 ± 0.17	n. f.	Wax, pear, leather
11	Ethyl phenylacetate	22.95	0.63 ± 0.05	0.63 ± 0.06	0.65	Honey, rose, fruity
12	Phenethyl acetate	23.45	70.9 ± 6.1	67.3 ± 5.4	0.25	Honey, sweet^6^
13	Ethyl cinnamate	27.76	2.32 ± 0.15	2.25 ± 0.11	n. f.	Fruity, fresh and sweet
14	Diisobutyl phthalate^*^	32.36	40.5 ± 3.8	31.4 ± 2.9	n. f.	Aromatic odor
15	Dibutyl phthalate	34.11	189 ± 9	137 ± 9	n. f.	Aromatic odor
	**Subtotal**		**1.02** **×** **10**^**3**^	**666**		
	**Subtotal (%)**		**37.5**	**34.7**		
**ACIDS**						
1	Hexanoic acid	23.86	11.6 ± 1.0	10.5 ± 0.5	0.42^5^	Cheese, rancid^7^
2	Octanoic acid	26.84	138 ± 8	129 ± 8	0.5^5^	Rancid, harsh, cheese, fatty acid^7^
3	Nonanoic acid	28.12	10.6 ± 0.7	–	0.5–0.8^2^	Cheese, waxy flavor^2^
4	n-Decanoic acid	29.38	77.3 ± 7.1	70.1 ± 6.7	1	Fatty, rancid
5	9-Decenoic acid	30.05	5.52 ± 0.23	–	1^8^	Fatty^8^
6	Dodecanoic acid	31.69	51.3 ± 5.4	–	1^5^	Dry, metallic, laurel, oily flavor^5^
7	Tridecanoic acid	32.75	17.5 ± 4.1	–	n. f.	Spice
8	Perillic acid (6CI)^*^	32.89	6.03 ± 0.34	3.70 ± 0.18	n. f.	Irritation
9	Tetradecanoic acid	34.00	113 ± 9	18.0 ± 3.0	n. f.	Odorlessness, flavor enhancer
10	Pentadecanoic acid	35.43	33.3 ± 2.8	9.18 ± 0.56	n. f.	Spice
11	n-Hexadecanoic acid	37.31	101 ± 9	34.0 ± 2.0	n. f.	Soil
12	Juniperic acid^*^	37.98	3.98 ± 0.21	–	n. f.	Musk
	**Subtotal**		**569**	**274**		
	**Subtotal (%)**		**20.9**	**14.3**		
**ALDEHYDES AND KETONES**						
1	Acetal	3.51	30.4 ± 2.0	–	n. f.	Aromatic odor
2	2-Octanone	11.77	9.53 ± 0.45	5.83 ± 0.33	0.25	Flowery, green fruit
3	4-Methyl-5H-furan-2-one	21.80	5.55 ± 0.28	–	n. f.	Pungent taste
4	Juniper camphor	29.24	29.7 ± 1.8	16.9 ± 2.6	n. f.	Irritation
5	Tridecanolactone	30.05	1.36 ± 0.05	–	n. f.	Spice
6	α-Cyperone	30.92	4.15 ± 0.31	–	n. f.	n. f.
7	Nootkatone	32.25	198 ± 13	153 ± 9	n. f.	Orange, sweet peel, woody
	**Subtotal**		**279**	**176**		
	**Subtotal (%)**		**10.3**	**9.17**		
**TERPENES**						
1	Linalool	18.40	13.8 ± 1.2	13.6 ± 2.1	0.025^9^	Flowery, fruity, muscat^8^
2	(–)-4-Terpineol	19.60	4.08 ± 0.29	3.29 ± 0.17	0.12	Flowers, clove
3	Citronellol	22.70	24.0 ± 3.0	24.6 ± 1.1	0.1^2^	Green lemon^2^
4	(Z)-Carveol^*^	23.77	3.77 ± 0.22	4.31 ± 0.33	n. f.	n. f.
5	Geranylacetone	24.08	7.86 ± 0.44	5.92 ± 0.39	0.06	Magnolia, green
6	β-Ionone	25.29	5.40 ± 0.26	–	0.00009^5^	Violet, sweet fruity^8^
7	Trans-nerolidol	26.64	2.91 ± 0.09	3.13 ± 0.16	0.7^7^	Sweet fruity, floral^5^
8	Farnesol	30.34	24.4 ± 2.0	25.6 ± 1.1	1	Sweet, floral, fragrant
	**Subtotal**		**86.2**	**80.5**		
	**Subtotal (%)**		**3.17**	**4.19**		
**OTHERS**						
1	Ethoxyethene	2.51	1.27 ± 0.03	–	n. f.	Ether
2	2-Methoxy-4-vinylphenol	28.51	21.4 ± 1.7	17.3 ± 2.0	0.04	Spicy, clove, curry powder
3	2,4-Di-tert-butylphenol^*^	29.79	5.64 ± 0.25	3.87 ± 0.19	0.20	Carbolic acid
4	Guaiazulene	37.77	1.80 ± 0.05	–	n. f.	n. f.
5	Squalene	41.34	3.55 ± 0.16	3.91 ± 0.23	n. f.	Pleasant, aromatic
	**Subtotal**		**33.7**	**25.1**		
	**Subtotal (%)**		**1.24**	**1.31**		
	**Total (mg/L)**		**2.72** **×** **10**^**3**^	**1.92** **×** **10**^**3**^		

*n. f., not found*.

Odor threshold and description taken from the following references:

1 Tao and Peng (2012);

2 Peng et al. (2013);

3 Sánchez-Palomo et al. (2010);

4 Tao et al. (2008);

5 Li et al. (2008);

6 Barata et al. (2011);

7 Li (2006);

8 Sun and Liu (2004);

9 Aznar et al. (2003). All data are expressed as the mean ± standard deviation of three replicates from two citrus wine samples.

* *The reference substances of methionol, ethyl trans-4-decenoate, diisobutyl phthalate, perillic acid (6CI), juniperic acid, (Z)-carveol, geranylacetone were not available. For these compounds the calibration curves of terpineol, ethyl 4-decenoate, dibutyl phthalate, 9-Decenoic acid, dodecanoic acid, cis-3-hexen-1-ol, α-ionone, respectively, were used*.

With regard to their concentration and odor thresholds, the odor-active components with odor active values (OAV) ≥1 included 1-pentanol, methionol, phenylethanol, ethyl acetate, ethyl butanoate, isopentyl acetate, ethyl hexanoate, methyl octanoate, ethyl octanoate, ethyl decanoate, ethyl phenylacetate, diethyl succinate, ethyl phenylacetate, phenethyl acetate, hexanoic acid, octanoic acid, Nonanoic acid, n-decanoic acid, 9-decenoic acid, dodecanoic acid, 2-octanone, linalool, (–)-4-terpineol, citronellol, geranylacetone, β-ionone, trans-nerolidol, farnesol, 2-methoxy-4-vinylphenol, and 2,4-di-tert-butylphenol. Table 4 shows different compounds with an OAV ≥ 1: with respect to the levels of alcohols, esters, acids, aldehydes, and ketones, terpenes, and others, either the individual concentration or the total concentration for CK4 was greater than that for sample #15.

**Table 4:** Total concentrations of compounds with an OAV ≥ 1 in CK4 and sample #15.

**No**.	**Compound**	**Concentration (mg/L)**	
		**CK4**	**Sample #15**
1	Alcohols	734	700
	Subtotal (%)	27.0	36.5
2	Esters	1.02 × 10^3^	666
	Subtotal (%)	37.5	34.7
3	Acids	569	274
	Subtotal (%)	20.9	14.3
4	Aldehydes and ketones	279	176
	Subtotal (%)	10.3	9.17
5	Terpenes	86.2	80.5
	Subtotal (%)	3.17	4.19
6	Others	33.7	25.1
	Subtotal (%)	1.24	1.31
	Total (mg/L)	2.72 × 10^3^	1.92 × 10^3^

### Sensory Tasting Analysis

Radial plots for each wine set are shown in Figure 2. These plots were used to identify potentially interesting sensory attributes. For set (a), the radial plot shows higher mean ratings for the attributes of color, condition (nose), purity (nose), intensity (nose), purity (palate), intensity (palate), length, and bitterness compared to those found for CK4. In contrast, we found a lower mean rating for the attributes of clarity, condition (palate), and balance for CK4. Therefore, the overall quality of CK4 citrus wine improved after treatment with the fining agent combination of sample #15. Furthermore, CK4 wine had a higher mean rating in terms of aroma characteristics like citrus, apple, banana, peach, floral, traditional Chinese medicine, licorice, spices, and bitter almond compared to that found in fining agent combination sample #15. Therefore, after fining, bitterness decreased, while characteristics of aroma and color (Granato et al., 2014) were lost to differing degrees.

Evaluation of our findings shows that several key characteristics of unfined wine and wine fined with different fining agents were different. A key finding related to DB is that the limonoid content was reduced by 36.24% in sample #15 compared to that in CK4. In addition, based on pH and total acidity measurements, the addition of fining agents definitively lowered the effect of acidity, and a combination of fining agents conferred a significant advantage in lowering the turbidity of citrus wine, as exemplified by the 59.13% reduction in turbidity found in sample #15, when compared to that found in CK4. Based on our thermal stability experiment, we found moderate turbidity in all samples. We also found that the retention rate of ascorbic acid was 65.59% lower in sample #15 than in CK4, indicating that the combination of gelatin and agar had a major effect on the adsorption and loss of ascorbic acid. From the thermal stability experiments, we also found that the retention rate of ascorbic acid decreased significantly, yet the rate was still higher for CK4 compared to that found in sample #15. During our extensive analysis, we found that the amount of pure precipitate in sample #15 was 0.15 g and that levels of ABTS and DPPH were slightly lower compared to those found in CK4, indicating that this combination of fining agents reduced the antioxidant capacity of the sample. The thermal stability experiments also showed that the antioxidative ability was greatly reduced in sample #15 compared to that in CK4. As shown in our comparison in Table 2, we found increased L^*^ and color brightness in sample #15 compared to CK4, and following the thermal stability tests, brightness generally decreased, although it remained higher in sample #15 compared to the control. Evaluation of a^*^ showed that sample #15 and CK4 were green, with greater color intensity found in sample #15, and following thermal stability testing, the color of sample #15 and CK4 turned red, with sample #15 appearing redder. Next, evaluation of the b^*^ values indicated that sample #15 and CK4 were yellow, with greater color intensity found in CK4, and that the color intensity for both increased following thermal stability testing. We also found from values of C^*^ that the color saturation of sample #15 and CK4 was low, and that the degree of color saturation increased following thermal stability testing. The values of H^*^ were all less than zero and, with respect to the general tendency of colors, sample #15 and CK4 appeared blue. Based on results from our thermal stability experiments, we found that the values of H^*^ were greater than zero, with a general color tendency of red for both sample #15 and CK4. Finally, based on values of E^*^, before and after heating stability experiments, a ΔE < 3 revealed no discernible difference between sample #15 and CK4.

As shown in Table 3 and Figure 2, it is evident that the number of components and the quantity of VCCs in citrus wine declined after fining from 50 to 36 and from 2.72 × 10^3^ to 1.92 × 10^3^ mg/L, respectively. We found that the number of alcohols (3 vs. 2), esters (15 vs. 14), acids (12 vs. 7), aldehydes and ketones (7 vs. 3), and terpenes (8 vs. 7) decreased in sample #15, relative to that in CK4. Table 4 shows that we found similar reductions in the total concentration of chemical compounds with an OAV ≥ 1 in CK4 and sample #15 for alcohols (734 vs. 700 mg/L), esters (1.02 × 10^3^ vs. 666 mg/L), acids (569 vs. 274 mg/L), aldehydes and ketones (279 vs. 176 mg/L), and terpenes (86.2 vs. 80.5 mg/L). We also found that the higher concentrations of phenylethanol in CK4 and sample #15 were 243 and 232 mg/L, respectively. Higher alcohols are secondary yeast metabolites and the optimal levels (below 300 mg/L) impart flowery, pollen, perfume, and rose characteristics (Li et al., 2008; Bartowsky and Pretorius, 2009).

### Verification Test Analysis

**Figure 3:**
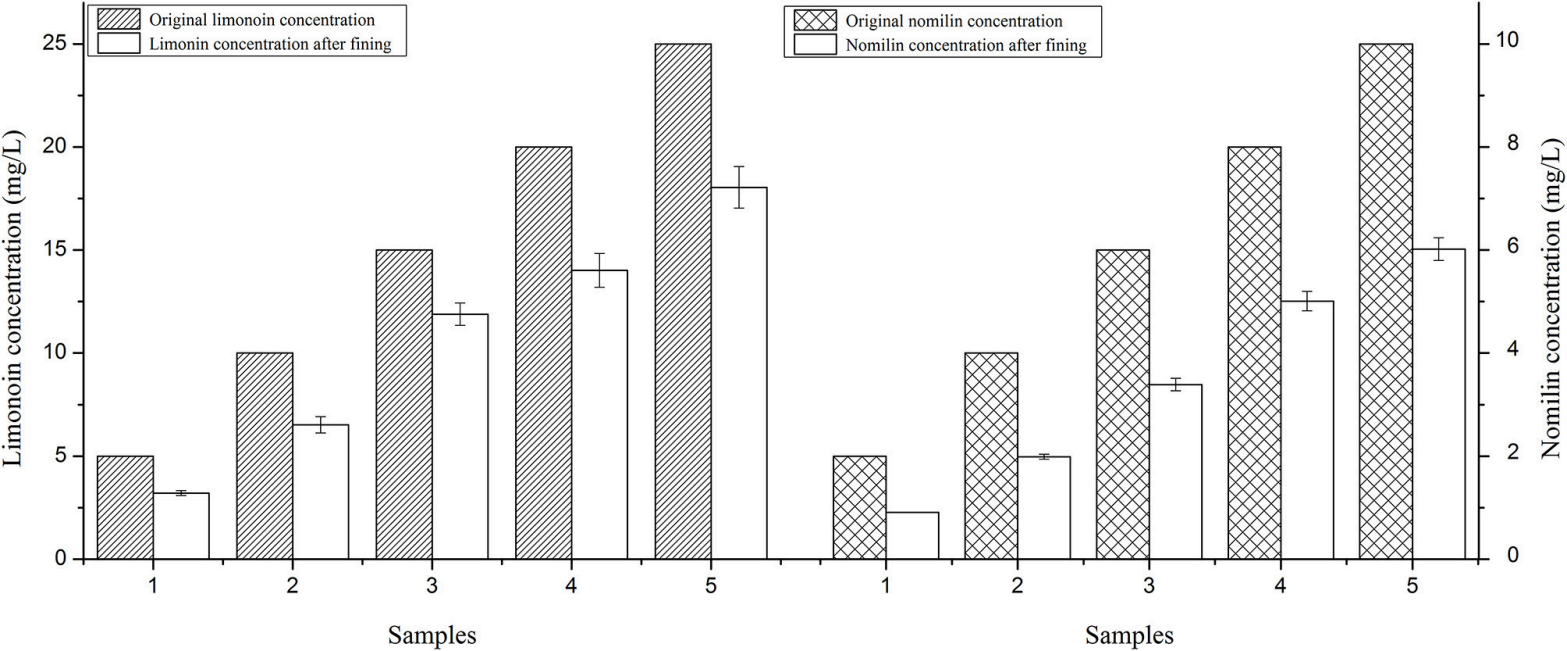
Limonin and nomilin concentrations in citrus wine model sample #15 treated with 125 mg/L agar and 30 mg/L gelatin.

As shown in Figure 3, after treatment with 125 mg/L agar and 30 mg/L gelatin as fining agents, the concentrations of limonin and nomilin in the citrus wine model systems decreased by differing degrees. It was further confirmed that using 125 mg/L agar and 30 mg/L gelatin as fining agents can reduce the concentration of limonoids in citrus wine.

**Figure 4:**
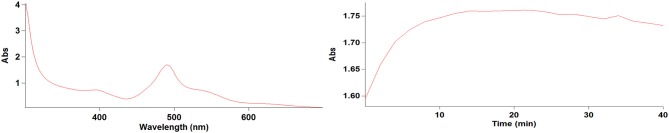
Full wavelength scan (nm) and selecting the best time (min) for measuring the absorbance (Abs).

Liang et al. (2013) found that the capacities of activated carbon and diatomite for adsorbing bitter substances was similar, while also possessing a clarifying role. Gelatin and agar have a positive and negative charge, respectively, and because of attractive effects, their interactions and coagulation can increase the surface area resulting in greater adsorption, thereby reducing the effects of bitter substances, such as total triterpenes (like limonoids). In addition, in the case of turbidity, gelatin and agar can interact with both positive and negative charges in the liquor, thus promoting greater overall clarification (Soto-Peralta et al., 1989). Importantly, gelatin (Fischerleitner et al., 2003) and agar can combine with phenolic substances, which in citrus includes naringin, hesperidin, and tannin with bitter citrus polyphenols (Balasundram et al., 2006), thereby neutralizing precipitation and reducing bitter taste. Finally, gelatin and agar lighten citrus wine by adsorbing pigment.

## Conclusions

Fining of fruit wine is an important part of the brewing process. In this study, we evaluated alternative uses of several adsorptive fining agents (agar, gelatin, chitosan, diatomite, bentonite, PVPP, and casein) at different concentrations with the aim of reducing the intensity of DB in citrus wine. Fining with almost all seven agents decreased the limonoid concentration, and our findings from single-factor, L${}_{3}^{4}$, and L${}_{2}^{3}$ fining experiments show that the concentration of limonoids decreased by 1.58–46.40%, 24.71–35.26%, and 19.65–27.78%, respectively, as we optimized the mixtures and concentrations of fining agents. We discovered that the combination of fining agents that provided the best results, as compared to control citrus wine samples, was 125 mg/L agar and 30 mg/L gelatin. Our findings consistently demonstrated that this combination of fining agents (sample #15 in this study) not only produced citrus wine with reduced DB intensity, but also clarified the wine body, achieved a larger net amount of precipitation, increased the retention rate of ascorbic acid, retained antioxidant activity, and maintained the special color of citrus wine. Finally, we also explored the sensory and aroma implications of using this combination of fining agents; however, a more intensive investigation into this aspect of fined citrus wine is warranted.

Because our study was conducted in an industrial setting, it provides an important link between the research and industry sectors, thereby allowing for a more effective translation of our results by commercial winemakers. Further investigation to determine how fining agents like gelatin, agar, and chitosan affect the concentrations of triterpenoids in citrus wine is required.

## Author Contributions

HL and JB conceived and designed the experiments. HW not only contributed reagents, materials, analysis tools, and guided the experiment, but also modified the language of the manuscript. JB carried out the experiments, analyzed the data, and wrote the manuscript. All authors have reviewed and approved the manuscript for publication.

### Conflict of Interest Statement

The authors declare that the research was conducted in the absence of any commercial or financial relationships that could be construed as a potential conflict of interest.
