# Supplementary material for: Delayed Bitterness of Citrus Wine Is Removed Through the Selection of Fining Agents and Fining Optimization
Source: Front Chem. 2019 Apr 2;7:185. doi: 10.3389/fchem.2019.00185 (PMC6454188; doi:10.3389/fchem.2019.00185)
Supplement: Supplementary file 1 [file Table_1.DOCX]

**实验说明及自愿参加实验声明书**

Experimental instructions and declaration of voluntary participation

研究负责人：李华教授

地点：中国 陕西 杨凌 邮编：712100 西农路22号 葡萄酒学院

研究目的：柑橘酒感官特征描述的研究

实验概况：你将应约来西农葡萄酒学院参加一次性实验。实验中您将观察、嗅闻和品尝柑橘酒并回答问卷。实验将持续约30min。

本次实验，我们将提供2款柑橘酒样品。请您在1. 观察; 2. 嗅闻; 3. 品尝酒样之后填写问卷 （每一项对应一张答卷）。 实验要求在品尝酒样之后将酒吐至吐酒桶内。如果实验前您的体内酒精浓度为0，正常情况下，本实验结束后，您的体内酒精浓度将不超过 0.2 g/L 血液。在实验结束，你离开实验室之前，我们将测量您体内的酒精浓度。如果您体内的酒精含量超过0.2 g/L的法定限值，您将暂时不能离开实验室。

资料的保密性：所收集的所有数据将作统计处理。只有进行本研究的科研人员可以接触到实验数据，并且实验数据只用于科学研究。如果实验结果将被发表，任何有关个人信息的数据将不会包含在内。依据1978年1月6日“信息和自由”修正案，您有知情权和修正权。若行使这项权利，请联系江滔老师（[tao.jiang@univ-lyon1.fr](mailto:tao.jiang@univ-lyon1.fr)）。

酬谢：为表达对您参与本实验的谢意，实验后赠送一板巧克力。

姓：....................名：...........地址：..............................................................................

我声明已了解了和知晓了这项研究和我有关的所有信息，并已得到了所有我口头要求想知道的信息。我是自由并自愿地参加所述的研究。我已被告知，我有权力随时终止参与此研究，同时，研究负责人也有权力在任何时候中止我参与这项研究。

我明白在每次品尝酒后， 我得将所品尝的酒吐至吐酒桶内。

我声明我年龄超过18岁，目前没有身孕，无酗酒问题，目前没有任何需要禁酒的治疗。

日期和申请人的签名（并手写以下内容：“我已阅读并理解上述文字中的所有信息”）

.........................................................................................................................................

日期及研究人员签名

.......................................................................................................................................
